# Different Approaches to Oxygen Functionalization of Multi-Walled Carbon Nanotubes and Their Effect on Mechanical and Thermal Properties of Polyamide 12 Based Composites

**DOI:** 10.3390/polym12020308

**Published:** 2020-02-03

**Authors:** Magdalena Kwiatkowska, Robert Pełech, Anna Jędrzejewska, Dariusz Moszyński, Iwona Pełech

**Affiliations:** 1 West Pomeranian University of Technology in Szczecin, Faculty of Mechanical Engineering and Mechatronics, 70-310 Szczecin, Poland; Magdalena.Kwiatkowska@zut.edu.pl; 2 West Pomeranian University of Technology in Szczecin, Faculty of Chemical Technology and Engineering, 70-322 Szczecin, Poland; Robert.Pelech@zut.edu.pl (R.P.); Dariusz.Moszynski@zut.edu.pl (D.M.); 3 Łukasiewicz Research Network–PORT Polish Center for Technology Development, 54-066 Wrocław, Poland; Anna.Jedrzejewska@port.org.pl

**Keywords:** polyamides, nanomaterials, functionalization, composites, mechanical properties, thermal properties

## Abstract

In this work the preparation of polyamide 12 (PA12) based composites reinforced with pristine and surface-modified carbon nanotubes is reported. A qualitative and quantitative evaluation of multi-walled carbon nanotube functionalization with oxygen containing reactive groups achieved by different procedures of chemical treatment is presented. Simple strong oxidative acid treatment as well as chlorination with subsequent chloroacetic acid treatment were applied. Carbon nanotubes (CNTs) were also subjected to chlorine and ammonia in gaseous atmosphere with small differences in after-ammonia treatment. Commercial COOH-functionalized carbon nanotubes were compared with nanotubes that were laboratory modified. The effect of CNT functionalization was evaluated basing on the improvement of mechanical and thermal properties of polyamide 12 composites prepared by in situ polymerization. It was found that high concentration of oxygen-containing functional groups on nanotube surface is not sufficient to improve the composite performance if the structure of carbon nanotubes is defective. Indeed, the best effects were achieved for composites containing nanotubes modified under mild conditions, seemingly due to a compromise between morphology and surface chemical structure.

## 1. Introduction

The unique properties of carbon nanotubes make them attractive for applications in many scientific and technological fields. Electronic structures, [1,2] polymer composites, [3,4,5], and biological systems [6] are some of the main applications that are intensively studied. In many applications it is necessary to tailor the chemical nature of the nanotube walls in order to take advantage of their unique properties. Using carbon nanotubes as a reinforcing component in polymer composites requires the ability to adjust the nature of nanotube walls in order to control the interfacial interactions between nanotubes and polymer chains. These interactions govern the load-transfer efficiency from the polymer to the nanotubes and hence the reinforcement efficiency. Several studies have been reported on the mechanical properties of nanotube polymer composites where nanotubes were used without surface modification [3,4,5]. These studies showed an increase in the elastic modulus of the composite at a relatively low nanotube concentration (down to 1 wt %). These findings show the potential of nanotubes as reinforcing components, especially if the interface between them and the polymer matrix is optimized.

In order to improve the efficiency of load transfer and make carbon nanotubes more compatible with a polymer matrix the covalent attachment of functional groups to the surface of carbon nanotubes (CNTs) is proposed. However, it must be noted that these functional groups might introduce defects on the walls of the perfect structure of nanotubes. These defects can lower the strength of the reinforcing component. Therefore, there is a trade-off between the strength of the interface and the strength of the nanotube filler. Oxidation in the liquid phase with agents such as nitric acid, hydrogen peroxide, sulfuric acid, potassium permanganate, and their mixtures [7,8,9] is the most common way of functionalizing carbon materials and leads to oxygen containing surface groups like alcohols/phenols, ketones, carboxylic acids, and their derivatives [10]. CNTs treatment with concentrated solutions of mineral acids [11] can be carried out by reflux, sonication, or microwave treatment. These procedures can last from a few minutes to several hours [12]. After oxidation the surface of CNTs has hydrophilic properties, which enhances the wettability of polar solvents. Moreover, oxygen functional groups can be used as anchoring sites for metal particles and large molecules [13]. The presence of carboxylic acid and hydroxyl groups on the nanotube surface is convenient because a variety of chemical reactions can be conducted with these groups.

Polymer composites containing carbon nanotubes have become one of the most extensively developed area of research due to high potential in production of light weight, high performance, and electrically conductive structural materials with a relatively low content of a nanofiller [14,15,16,17,18]. In particular, CNT composites based on thermoplastics are of great interest for industry, since a variety of processing methods offers a broad range of forms and shapes of potential composite final products [19,20,21,22,23,24]. Among the large group of engineering thermoplastics, polyamides occupy one of the highest positions due to their diversity in physical properties. This was determined by both the COHN to CH_2_ group ratio and the number of CH_2_ groups in repeat units of a polymer chain [25,26]. Basically, melting point, crystallinity, and water absorption of different types of aliphatic polyamides strongly depend on these factors. Good mechanical, thermal, and electrical performance of polyamides arises from strong intermolecular cohesion due to hydrogen bonding between adjacent polymer chains. All of these features have made polyamides expand their applications in a variety of fields, ranging from precision injection molded parts, automotive, transportation, electrical engineering, medical instrumentation, up to technical fibers. Numerous researchers have also reported on the effect of carbon nanotubes on polyamide-based composites [27,28,29,30,31]. Considering the approach to chemical modification of carbon nanotubes and expected functional groups at the CNT surface in this study, polyamides seem to be a suitable polymer matrix used to evaluate the effectiveness of modification. Applying in situ polymerization in the presence of functionalized nanotubes for composite preparation gives a chance for functional groups to grow polymer chains in chemical reactions to occur. Such effects have been observed in composites based on epoxy [32], polyvinyl alcohol (PVA) [33], polycarbonate (PC) [34], and others [35]. Polyamide 12 is typically obtained via ring-opening polymerization of lauryl lactam, which proceeds in two steps. First opening of lactam rings to 12-aminododecanoic acid and its addition to oligoadducts is induced by the presence of water, whilst in the second step polycondensation of oligoamides proceeds and growing polymer chains are terminated by amine groups with dicarboxylic acid reactions [25]. In our study we assumed that some growing polymer chains may also be terminated by reactive functional groups attached on nanotube surface when encountered in the reaction mixture. Moreover, CNT surface groups can contribute to hydrogen bonding increasing strength of interphase interactions in the composite. Thus, both chemical and physical interactions between two phases in polyamide 12 (PA12) composites were expected, which is the key issue for good performance of a composite.

The purpose of this study was a qualitative and quantitative evaluation of CNT functionalization with oxygen functional groups achieved by different procedures as well as the effect of functionalized carbon nanotubes on polyamide 12-based composites’ performance. The morphology of CNT was studied using high-resolution transmission electron microscopy, and Raman spectroscopy, whilst the surface functionalization/composition was analyzed using X-ray photoelectron spectroscopy. For the characterization of PA12 composites the thermal (differential scanning calorimeter (DSC) and thermo-gravimetric analysis (TGA)) and thermo-mechanical (DMTA) analyzes were performed, the chemical structure was confirmed by FTIR technique, the mechanical properties were assessed in the static tensile tests, whilst the state of CNT dispersion in composites was evaluated using scanning and transmission electron microscopy. Commercial COOH– modified multi-walled carbon nanotubes were also used as the nanofiller in order to compare with nanotubes that were laboratory treated. 

## 2. Materials and Methods 

### 2.1. Chemical Functionalization of Carbon Nanotubes 

Commercially available multiwalled carbon nanotubes NC7000, Nanocyl S.A., Belgium (denoted as NC) were the pristine material subjected to chemical functionalization. According to the supplier their average diameter and average length were 9.5 nm and 1.5 µm, respectively. Carbon purity using thermogravimetric method was higher than 90%. The material was submitted to different modification procedures in order to attach oxygen functional groups on the surface. 

The sample denoted as NC_01 consists of commercial multi-walled carbon nanotubes under the trade name of NC3101 (Nanocyl s.a. Belgium) with carboxyl groups attached on the surface. According to the supplier their average diameter and average length were 9.5 nm and 1.5 µm, respectively. Carbon purity determined using thermogravimetric method was higher than 80%. The amount of carboxyl groups determined using thermogravimetric method was about 8%. Other CNT samples denoted from NC_02 to NC_05 were laboratory treated as follows: 

Carbon nanotubes denoted as NC were placed in a nitric acid (HNO_3_) and boiled for 24 h under reflux to obtain acid-modified multi-walled carbon nanotubes. After the treatment, CNTs were boiled in distilled water and the process was repeated three times. The resulting material was filtered and dried in 110 °C for 24 h. The sample was denoted as NC_02. 

The sample denoted as NC_03 was chlorinated according to a procedure described in [36]. Subsequently, the obtained material was submitted to hydrolytic dechlorination. The sample was placed in a flask, quenched with sodium hydroxide solution, and boiled for 1 h under reflux. Next the content of the flask was cooled and filtered. The precipitate was washed with 1 M HNO_3_ and distilled water to remove alkali residuals. Finally, the sample was boiled in distilled water until neutral pH was reached, which indicated the removal of leaching reagents. In order to obtain carbon material with carboxy functional groups on the surface, the sample after hydrolytic dechlorination was placed into a flask together with chloroacetic acid. 1 M NaOH was introduced drop wise. All the content was boiled under reflux for 1 h. Next the mixture was cooled to the room temperature and filtered. The obtained material was washed using water with the addition of 5 M HNO_3_, filtered, and dried at 110 °C for 24 h. 

The samples denoted as NC_04 and NC_05, were chlorinated according to a procedure [36] and submitted to reaction with ammonia. The sample after chlorination was placed in a reactor. When the temperature reached 400 °C, ammonia was introduced, and the reaction was continued for one hour. Then residual gases were evacuated using a vacuum pump as long as an ambient temperature was reached. Finally, the sample was boiled in distilled water and dried in a vacuum oven at the temperature of 110 °C for 24 h. In order to obtain the sample denoted as NC_04 carbon material after reaction with ammonia was placed into a flask filled with water, chloroacetic acid, and sodium hydroxide. All the content was mixed with a magnetic stirrer and boiled under reflux for 2 h. Next the sample was filtered, boiled in distilled water, and finally dried in a vacuum oven at the temperature of 110 °C for 24 h. In order to obtain the sample denoted as NC_05 carbon material after reaction with ammonia was placed into a flask filled with dioxane. Methyl chloroacetate and anhydrous AlCl_3_ were added to the mixture. All the content was mixed with a magnetic stirrer and boiled under reflux for 2 h. The filtered carbon material was washed with dioxan in Soxhlet extractor and finally dried in a vacuum oven at the temperature of 110 °C for 24 h. 

### 2.2. Preparation of PA12/Carbon Nanotube Composites 

Nanocomposites based on PA12 and modified multi-walled carbon nanotubes were prepared by in situ synthesis via ring-opening polymerization of laurolactam (LL, 12-Aminododecanolactam, 98%, SAFC, Buchs, Switzerland) in the presence of sebacic acid (SA, >95%, Sigma Aldrich) as the molecular weight stabilizer. Carbon nanotubes were first dispersed using ultrasonication and ultra-high mechanical stirring in a solution of lauryl lactam in methanol, then alcohol was evaporated to let lactam re-crystallize. The polymerization process was carried out in a steel chemical reactor of 1 dm^3^ in volume (Autoclave Engineers, Division of Snap-title, Inc., Erie, PA, USA) in the presence of the nanofiller under continuous mixing and under appropriate temperature and pressure conditions. The progress of polymerization was monitored via rise in the stirrer torque due to increasing viscosity of the reaction mixture. At the end of the process a nanocomposite in the molten state was extracted from the reactor using compressed nitrogen, pelletized, and methanol washed to extract unreacted monomer residues. Details concerning the synthesis parameters were described in [37]. This way composites containing 0.35 wt % of different CNT samples were obtained and subjected to further characterization. Testing samples, including the tensile tests, in the form of dog-bone shaped pieces, with a total length of 60 mm, a rectangular cross section of 2 by 4 mm^2^, and a gage length of 20 mm were prepared by injection-molding according to ISO 37:2005 type 3. 

### 2.3. Characterization of Carbon Nanotubes 

The morphology of carbon nanotubes was studied using high-resolution transmission electron microscope (HRTEM)—FEI Tecnai G2 F20 STWIN, US. Raman spectroscopy (Renishaw InVia Raman Microscope spectrometer, Gloucestershire, UK) with excitation laser lines of 785 nm (1.58 eV) was used to characterize the defect ratio in carbon nanotube structure. Surface functionalization was analyzed using X-ray photoelectron spectroscopy (XPS). XPS spectra were acquired with a SES 2002 spectrometer operating at constant transmission energy (Pass Energy = 50 eV) using Al Kα radiation (*hν* = 1486.6 eV). The instrumental resolution, as evaluated by the full-width at half maximum (FWHM) of the Ag 3d_5/2_ peak, was 1.0 eV. Samples were placed into a grooved sample holder. The analysis chamber during experiments was evacuated to more than 1 × 10^−9^ mbar. The surface composition of the samples was obtained on the basis of peak area intensities using the sensitivity factor approach and assuming homogeneous composition of the surface layer. 

### 2.4. Characterization of Polymer Composites 

The state of CNT dispersion in the polymer matrix was investigated using high-resolution scanning electron microscopy (SEM, Hitachi SU-70, Tokyo, Japan) on cryo-fractured in liquid nitrogen samples after injection molding, and the exposed surfaces were sputtered with a thin gold film in vacuum.

The weight-average molecular weight of the composite matrix M_w_ was calculated according to Mark–Houwink equation as follows [25]:

(1) $$\left[\eta \right]=4.6\cdot 10^{-4}{\left(M_{w}\right)}^{0.75}$$

The intrinsic viscosities [*η*] of the samples were determined using a capillary Ubbelohde viscometer (type Ic, *K* = 0.03294) at 25 °C using 0.5 g/dL concentration of polymer in m-cresol (99%, Sigma Aldrich) solutions. Before the measurements, solutions were filtered to remove carbon nanotubes.

The chemical structure of composites was confirmed by attenuated total reflectance-Fourier transform infrared spectroscopy (ATR-FTIR) analysis using Tensor-27 (Brucker, Ettlingen, Germany) spectrophotometer equipped with a germanium crystal ATR accessory. The spectra were recorded in a wave number range of 4000–600 cm^−1^, and normalized. 

A differential scanning calorimeter (TA Instruments Q 100, New Castle, DE, USA) was employed to characterize thermal transitions and the crystalline structure of polymer matrix. Samples were analyzed in heating–cooling–heating cycles in the temperature range of −30 to 220 °C with the standard heating rate of 10 °C/min. The mass degree of crystallinity (*X_c_*) was calculated according to:

(2) $$X_{c}=\frac{\Delta H_{m}}{\Delta H_{m}^{0}}100\%$$

where $\Delta H_{m}^{0}$ is the theoretical value of enthalpy for 100% crystalline PA12 $\Delta H_{m}^{0}$ = 209.2 J/g [25].

The thermal stability of nanocomposites in both thermo–oxidative and neutral atmosphere was analyzed using thermo-gravimetric analysis (NETZSCH TG 209F1 Libra). The measurements were carried out with the heating rate of 10 °C/min up to 800 °C.

Dynamic mechanical thermal analysis was performed in DMA (Q800 model, TA Instruments, New Castle, DE, USA) operated in multi–frequency strain mode. The temperature range analyzed was −100 to 150 °C at a constant frequency of 1 Hz.

Tensile tests were performed on a universal testing machine (Instron 5566, Norwood, MA, USA) equipped with an optical long travel extensometer. The injection-molded samples were deformed with the speed of 20 mm/min using 5 kN force transducer, and a grip distance of 20 mm at room temperature. A minimum of six tests were made for each sample. The tensile properties of composite materials were determined according to ISO 527-1,2:2012, and the Young’s modulus was calculated from the slope of stress–strain characteristic at a very low strain range. The Bluehill 2 software (version 2, Instron, Norwood, MA, USA) was used to collect the data. 

## 3. Results

### 3.1. Analysis of Carbon Nanotube Functonalization 

**Figure 1:**
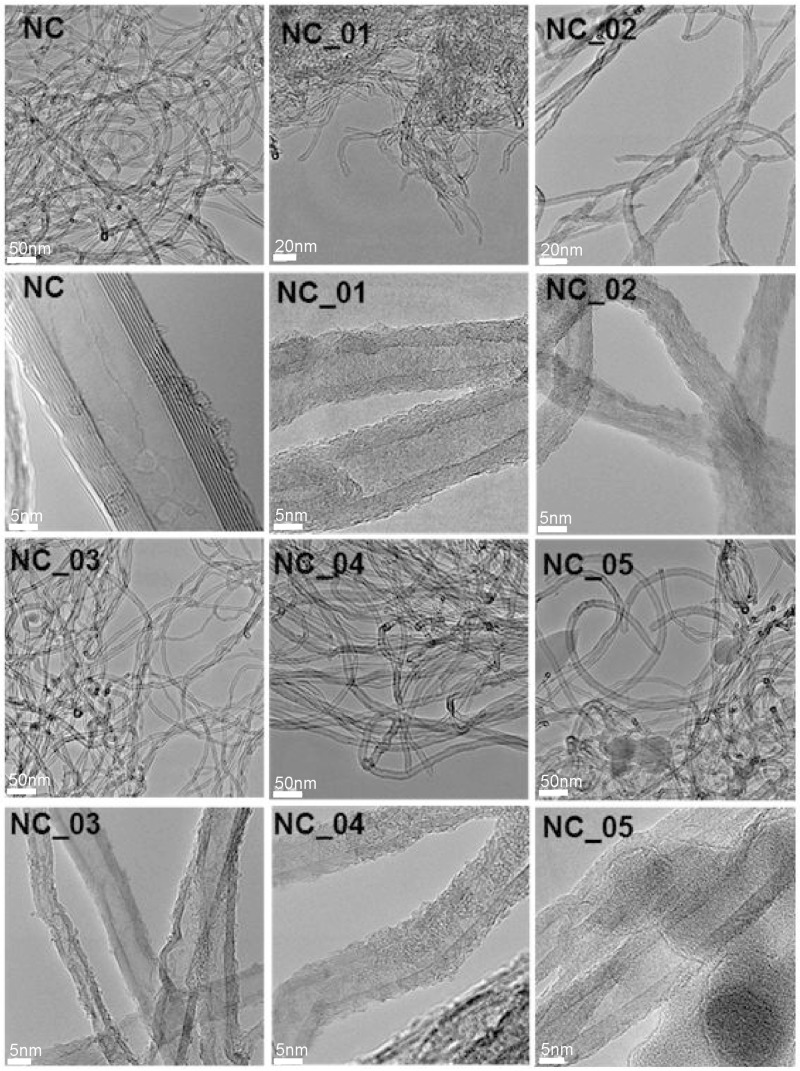
TEM images of raw (carbon nanotubes NC7000, Nanocyl S.A., Belgium (NC)) and modified (NC_01, NC_02, NC_03, NC_04, NC_05) carbon nanotubes.

Figure 1 presents TEM images of pristine (NC) and functionalized (NC_01, NC_02, NC_03, NC_04, and NC_05) carbon nanotubes. As can be seen, all the functionalized carbon nanotubes are characterized by high surface roughness in comparison to pristine material (NC). The surface roughness can indicate that graphitic carbon was partially damaged as a result of functionalization and/or oxidation processes [38]. With use of TEM technique it is not possible to distinguish functional groups. However, the roughness could be taken as a signature of surface deterioration of CNTs due to chemical treatment. The functionalization reaction disrupts the sp^2^ carbon network of graphitic CNTs, so it may be responsible for the roughness of CNT surfaces [39]. Comparing the images, we can conclude that the highest surface roughness characterizes samples obtained during the reaction with nitric acid (wet oxidation) (NC_02). The treatment of CNTs with strong oxidizing agents causes severe etching of the graphitic surface of the material, leading to tubes with a population of disordered sites [40]. Whereas the dark spots visible at the images of the sample NC_05 can refer to the presence of aluminum oxide which was not flushed. The presence of aluminum oxide on the surface of this material is also confirmed by the XPS method described further.

**Figure 2:**
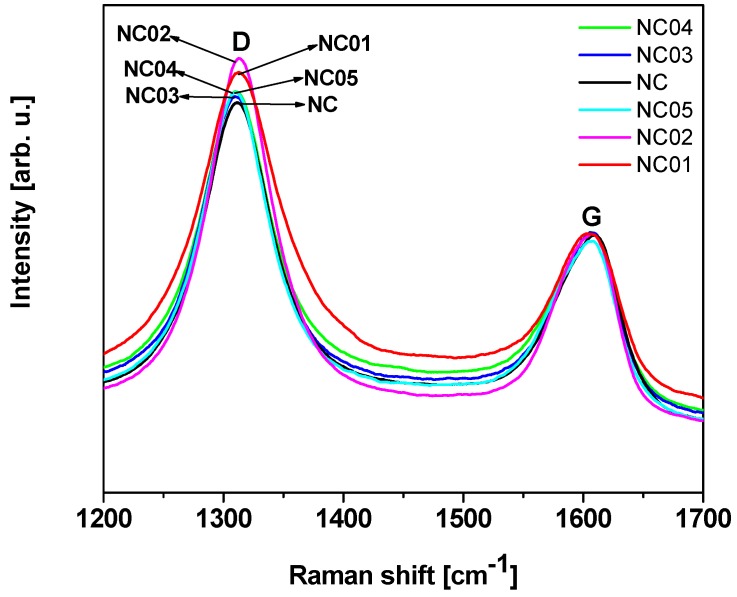
Raman spectra of pristine and functionalized carbon nanotubes.

Raman spectroscopy provides information related to the structural changes of the nanotubes and can be a direct evidence of the chemical functionalization [41,42]. The Raman spectra collected for pristine (NC) and functionalized (NC_01, NC_02, NC_03, NC_04, and NC_05) carbon nanotubes are shown in Figure 2. Two major bands were observed: D band, resulting from the formation of sp3 bonded carbon atoms, at approximately 1300 cm^−1^, and G band, that relates with the tangential mode vibrations of the sp^2^ bonded carbon atoms, at approximately 1600 cm^−1^ [43,44]. The D band is a double-resonance Raman mode affected by defects in the graphene structure. This band together with the G band can be used for material characterization to probe and monitor structural modifications of nanotube sidewalls that come from the introduction of defects and the attachment of different chemical species [45]. The degree of functionalization can be quantified using the D to G band intensity ratio (ID/IG), which provides an estimation of the ratio of sp^3^/sp^2^ carbon atoms in multi-walled carbon nanotubes [44,46,47]. The calculated values of the intensity ratio ID/IG are presented in Table 1. All the functionalized materials are characterized by an increase in the ID/IG intensity ratio compared to the unmodified sample. Similar results were observed by Yinglong et al. [48]. They claimed that the increase of the intensity ratio ID/IG for single-walled carbon nanotubes functionalized with hydroxyl groups in comparison with raw carbon nanotubes proves the presence of covalently-functionalized groups on CNTs –OH. Rong Tian et al. [49] observed the increase of the ID/IG intensity ratio for functionalized single-walled carbon nanotubes with alcohols under microwave irradiation in comparison with raw material, which indicates that a number of sp^2^ hybridized carbons have been converted to sp^3^ hybridization carbons as a result of the connection of the functional groups to the surface [49]. Maofei et al. [50] noted the highest changes in the intensity ratio ID/IG for multi-walled carbon nanotubes treated with nitric acid and this fact was explained as the introduction of new defects as well as changes in the geometry of CNTs.

In the case of our samples the highest value of the ID/IG intensity ratio characterized a sample modified with nitric acid (NC_02). Osswald et al. [51] claimed that it is associated with the increased number of defects on multi-walled carbon nanotube walls and edges introduced during oxidation. Results obtained from Raman spectroscopy confirm TEM observations, where modified samples are characterized by higher surface roughness in comparison with pristine carbon nanotubes and the highest surface roughness was assigned to NC_02 material.

The surface composition of carbon nanotubes was examined by means of X-ray photoelectron spectroscopy. On the surface of all samples carbon and oxygen were identified. Samples NC_03, NC_04, and NC_05 also contained chlorine. On the surface of samples NC_02 and NC_ 04 traces of nitrogen were detected. Sample NC_05 contained an addition of aluminum. The quantitative elemental composition of these surfaces is calculated upon X-ray photoelectron spectroscopy data and given in Table 2. The main component of all sample surface is carbon, which constitutes between 92 and 97 percent of atoms composing the surface. The only exception is sample NC_05 which was doped with aluminum compounds during functionalization procedure. As a result, there is a substantial amount of aluminum atoms on the surface and a considerable increase of oxygen and chlorine content is observed. Taking into consideration the samples NC_01 to NC_04 the highest oxygen content is observed on the surface of sample NC_01 (7.3 atomic %) and NC_02 (6.4 atomic %) and it decreases to 2.5 atomic % for sample NC_04. 

**Figure 3:**
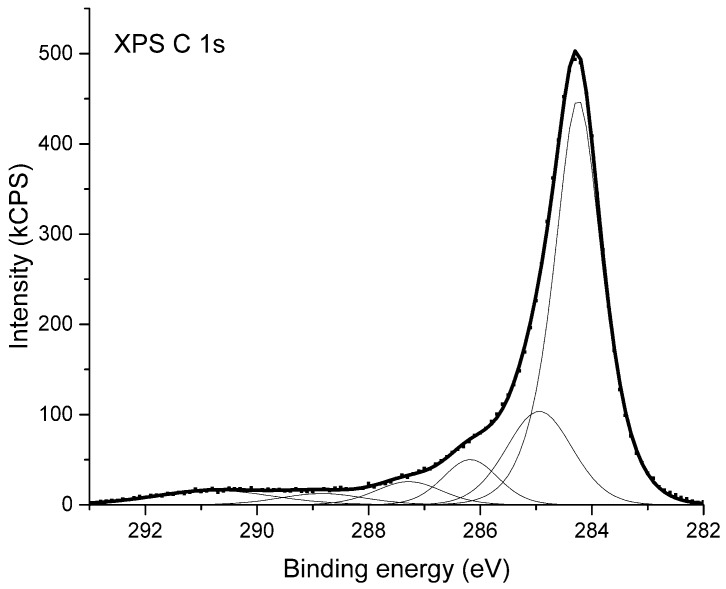
X-ray photoelectron spectrum (XPS) of carbon nanotubes from sample NC_01.

High-resolution X-ray photoelectron spectra were used to examine the chemical state of the substances composing the surface of the studied materials. In Figure 3 the XPS C 1s spectrum coming from sample NC_01 is shown. The XPS C 1s peak has a maximum at the binding energy of 284.3 eV. It was deconvoluted into six components shown as thin lines below the envelope of X-ray photoelectron spectroscopy data. The deconvolution is based on the model presented in [52]. Considering the components from the lowest binding energy one can attribute the given peak to the following chemical bindings of carbon atoms: Position 284.3 eV corresponds to non-functionalized carbon atoms located in graphitic rings; position 285.0 eV is attributed to all other non-functionalized sp^3^ carbon atoms, bonded either with second carbon or with hydrogen atoms; position 286.2 eV is ascribed to a group of differently bonded carbon atoms linked to one atom of oxygen i.e., functional groups such as C–O–C or C–OH; position 287.3 corresponds to functional groups such as C=O or O–C–O; position 288.8 eV is attributed to carbon atoms indicated by asterisk in the functional groups like C–O–C*=O or HO–C*=O; and position 290.8 eV is attributed to shake-up structure caused by the π→π*-transition in graphite rings [53].

The analysis of XPS C 1s spectrum from NC_01 sample indicates that the main component of the surface are carbon atoms located in graphitic rings. A part of carbon atoms is also bound to oxygen atoms and forms different carbon–oxygen moieties. The XPS C1s spectra of other samples under consideration are virtually identical.

**Figure 4:**
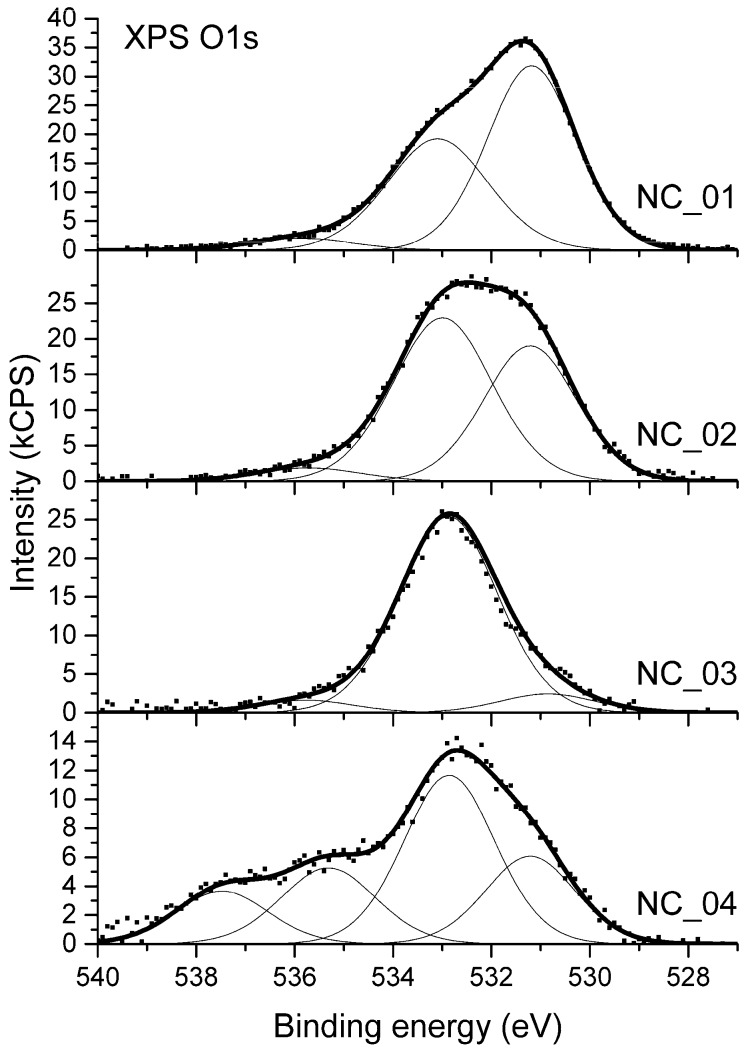
XPS O 1s spectra from carbon nanotubes.

The analysis of high-resolution XPS O 1s spectra was applied to evaluate the ratio between C–O and C=O bonds present on the surface. XPS O 1s spectra acquired for samples NC_01 to NC_04 are shown in Figure 4. The spectrum from sample NC_05 is not shown since a severe differential changing of the sample surface was observed. Due to this effect, proper binding energy calibration was impossible. Moreover, high-resolution spectra of all elements identified for that sample were distorted as if the peaks from charged surface were overlapped by peaks from a part of surface which was not charged. Therefore, the X-ray photoelectron spectrum from sample NC_05 was only used for quantitative analysis of surface elemental composition. 

The XPS O 1s spectra differs noticeably between samples as shown in Figure 4. Prior analysis of XPS C 1s components indicates the presence of different oxygen-bearing functional groups such as C–O–C, C–OH, C=O, C–O–C*=O, or HO–C*=O. Regarding oxygen atoms two general chemical states can be distinguished in all the mentioned functional groups: Singly-bonded oxygen (C–O) and double-bonded oxygen (C=O). Therefore, the deconvolution of XPS O1s spectra in Figure 4 was based on the two major components: The first one for C=O bonds, which were settled at the position of 531.0 ± 0.1 eV and the second one for C–O bonds at the position of 533.0 ± 0.1 eV. A third component was also required to be added, which is representative to adsorbed water and is usually positioned in the range of binding energy of 535.4–535.8 [54,55]. A full deconvolution of O 1s spectrum acquired for sample NC_04 required an additional component, which was located at the binding energy of 537.5 eV. Its attribution is uncertain and further in the text it is denoted as “satellite”.

Application of the deconvolution model enables one to calculate the fractional composition of chemical states of surface oxygen. Results of these calculations are shown in Table 3. 

Considering the proportion between singly-bonded oxygen C–O and double-bonded oxygen C=O functional groups, the samples can be divided into two groups. On the surface of NC_01, NC_02, and NC_04 samples, the fraction of C=O bonds is relatively high. Regarding XPS C 1s spectrum (Figure 3) isolated C=O bonds as well as –COOH moieties can be present on the surface. The prominent intensity of C=O component in XPS O 1s spectra acquired for these three samples proves that the surface is abundant in carboxyl groups. On the surface of sample NC_03 the component in XPS O 1s spectrum coming from C–O bonds prevails. This indicates that the surface of this sample is enriched with hydroxyl groups. 

### 3.2. The Effect of Carbon Nanotube Functinalization on Performance of Polymer Composites 

The effect of oxygen functionalized carbon nanotubes, obtained in different procedures, was investigated regarding polyamide 12 based composite performance. Commercially modified multi-walled carbon nanotubes were also used as the nanofiller in order to compare with nanotubes that were laboratory treated. The characteristics of the PA12 composite filled with untreated multi-walled carbon nanotubes can be found elsewhere [37]. All composites contained the same amount of CNTs, i.e., 0.35 wt %. 

**Figure 5:**
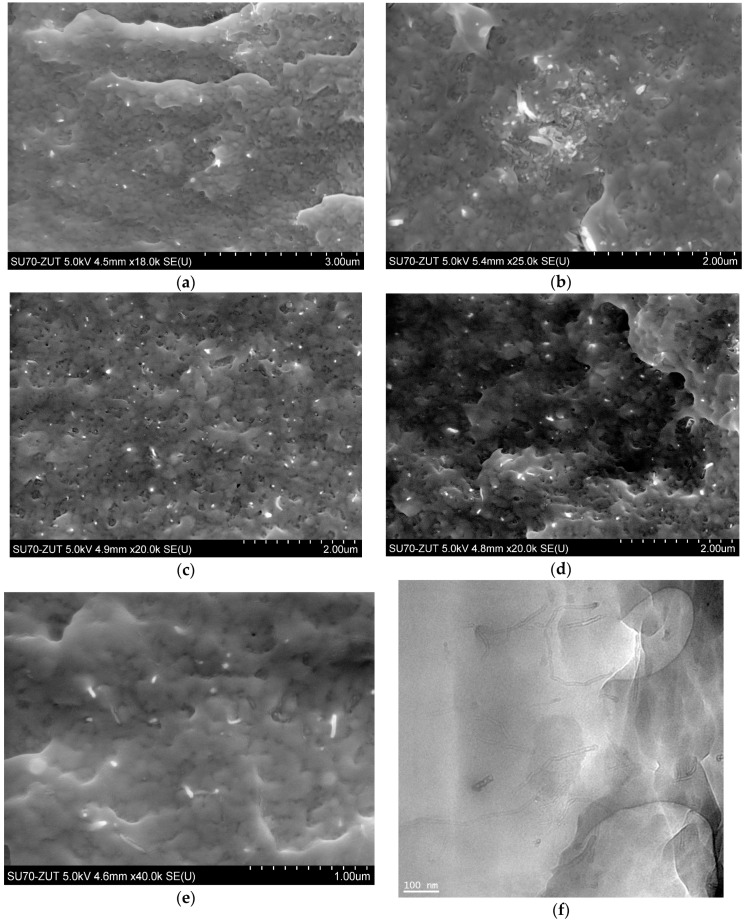
SEM micrographs of composites: (**a**,**b**) PA/NC_01, (**c**) PA/NC_04, (**d**) PA/NC_05, (**e**) PA/NC_02, and TEM micrograph of NC_04 sample (**f**).

The in situ synthesis of composites was performed via ring-opening polymerization of lauryl lactam preceded by dispergation of nanotubes in a monomer/alcohol solution using mechanical stirring and ultrasonication. Based on the proceeding reactions it was assumed, that COOH– and OH– functionalized carbon nanotubes dispersed in the reaction mixture statistically increase the chance for chemical reactions between functional and amine, amide, or carboxylic groups of growing macromolecules, as it happens in the case of sebacic acid terminating polymer chains. In addition, surface groups, which did not react, may create hydrogen bonds between nanoparticles and macromolecules increasing strength of interfacial interactions as well. In this manner both chemical and physical interactions between two phases in PA12 composites were expected, which is the key issue for good performance of composites. Results obtained in this study suggest however, that not only chemical treatment (therein a procedure of functionalization and functional group content), but also the macroscopic form of carbon nanofiller introduced into the polymer, greatly influence composite morphology and characteristics. It is first evident, when the state of nanotube distribution in a polymer matrix is analyzed. NC_01, as received from the supplier, had a form of highly non-uniform powder with numerous clods visible to the naked eye. Application of mechanical forces and ultrasounds was insufficient to break the existing agglomerates of macroscopic sizes in this case, which was proved by SEM analysis. Figure 5a,b depicts PA/NC_01 surface fractures, which are dominated by large clusters of nanotubes or areas of much lower CNT concentration indicating very poor dispersion of nanotubes. Significantly better results were achieved for composites containing laboratory functionalized nanotubes. Careful crumbling of nanomaterials after chemical treatment and subsequent dispergation in a monomer bring much more benefits in distribution of nanofillers, as visible in SEM micrographs (Figure 5c–e). In all composites, nanotubes are mostly separated and practically fully embedded in the polymer matrix, only bare CNT endings uncovered during fracturing can be perceived. It is also confirmed by TEM micrograph of NC_04 sample (Figure 5f). It suggests that most nanotube agglomerates were destroyed before polymer synthesis at the dispergation stage, and growing polymer chains only assisted in further separation. The (so-called) “pulled–out” effect is practically not observed, when there is a good wettability of carbon nanotubes by the polymer matrix. In general, microscopic observations confirm high effectiveness of the in situ method for PA12-based composite preparation if the state of CNT dispersion is considered. They also indicate that nanotube distribution is practically not affected by the results of functionalization procedure or functional group content, but mainly by the state of bulk CNT powder and care in its preparation. On the other hand, nanotubes wetted by polymer may result in possible chemical bonding between two phases of composite during polymerization. It can be attributed to efficient CNT modification, and the procedure of chemical treatment gains in importance, when composite mechanical performance is taken into consideration. 

**Figure 6:**
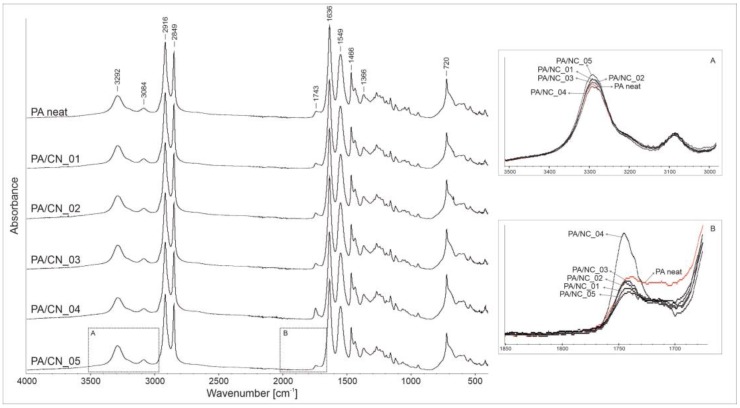
The FTIR spectra of polyamide 12 (PA12)/NC composites.

The FTIR analysis was performed in order to confirm the real chemical structure of the investigated composites as well as possible phase interactions between constituents. Figure 6 presents the spectra recorded for all materials with the absorption regions revealing differences among samples, zoomed in boxes. In general, both the PA12 homopolymer and its composites disclose the absorption peaks characteristic for polyamides, namely those corresponding to amide group: At 3292 and 3084 cm^−1^ (stretching vibration of N–H bonds), 1549 cm^−1^ (stretching vibration of C–N bonds), and 1635 cm^−1^ (stretching vibration of C=O bonds), as well as those attributed to aliphatic part of the macromolecule: At 2916 and 2849 cm^−1^ (stretching vibration of C–H bonds in CH_2_ groups), 1466 cm^−1^ (C–H bond bending), and 1366 and 720 cm^−1^ (C–H bond rocking). The wavenumber positions of all identified reflections are the same for investigated materials, what confirms that the presence of CNT in reaction mixture does not affect the polymerization process or chemical structure of the polymer matrix. However, some gentle differences in intensities, observed in 3500–3000 and 1750–1700 cm^−1^ absorption regions, may indicate physicochemical interactions between constituents in composites. Variations in intensity of the 3292 cm^−1^ absorption band (box A) may be associated with interactions between N–H bonds (of the amide group) and numerous oxygen-containing reactive groups on nanotubes’ surface. More visible changes are detectable in the carbonyl group absorption region (box B). A decreasing intensity of reflections at around 1700 cm^−1^, observed only for composites, might indicate that the carboxylic end groups in polymer chains have undergone some chemical reactions, most likely with the hydroxylic groups on CNT’s surface. The exact determination of the type of nanofiller–polymer chemical interactions requires more advanced structure analysis, nevertheless the identified alterations in FTIR spectra indicate for physicochemical interactions as highly probable. 

**Figure 7:**
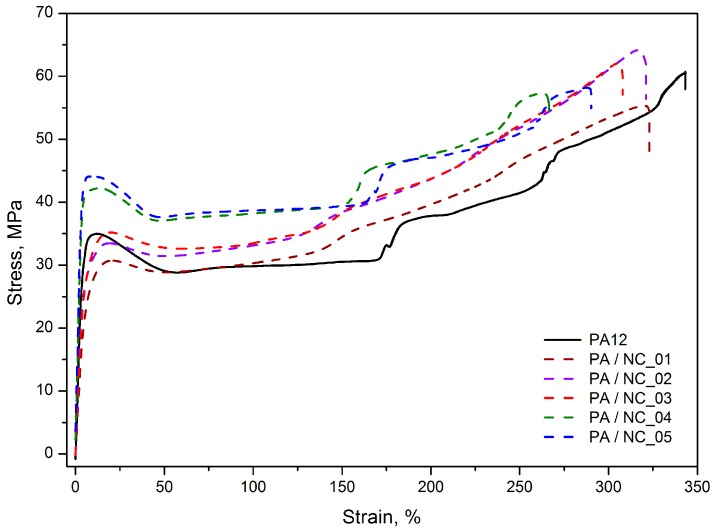
Representative tensile stress–strain characteristics of PA12/NC composites.

Although the tensile stress–strain characteristics obtained for PA12/CNT composites, presented as representative in Figure 7, are similar in the shape, with a clear yield point, then a gradual increase in stress and strain as well as the necking effect, clear differences in the average values of yield stress (σ_y_), stress at 200% of strain (σ_200%_), and Young’s modulus (*E*) are observed depending on the nanotube functionalization method (Table 4). The tensile tests display the highest improvement in mechanical strength for PA/NC_04 and PA/NC_05 composites. The increase up to 26% in σ_y_ and 29% in σ_200%_ for only 0.35 wt % of nanotube content was achieved. It should be mentioned here, that when investigating mechanical strength of polymers with a clear yield point, the stress at yield is, in fact, more important than the stress at break, because it determines the level of material’s elastic deformation and when exceeding it a sample changes its dimensions permanently. These composites have also the highest stiffness with the E modulus upswing up to 39%. Such improvement in mechanical performance (also noticeable if the tensile tests parameters for composites containing pristine NC are considered) suggests an effective cooperation of two phases in a composite or increase in crystallinity of polymer matrix in the presence of CNTs. However, since the crystallinity degrees of PA/NC_04 and PA/NC_05 are only slightly higher than that of neat PA12 (Table 5), the enhancement of strength could be attributed to the effect of interface interactions between composite constituents due to the chemical bonding. Indeed, as concluded from the FTIR analysis, the presence of OH– and COOH reactive groups attached to CNT surface increases the probability for chemical reactions between nanofillers and growing polymer chains during polymerization, for example by terminating macromolecules. It would result in reducing the molecular weight of polymer matrix, and a drop in composite deformation ability. All these effects are observed in the case of PA/NC_04 and PA/NC_05 samples in this study (Table 3). Also, for the PA/NC_04 composite the highest intensity of reflection in the carbonyl group, absorption region was observed suggesting the chemical reactions to take place.

Interestingly the other composites, excluding PA/NC_01, exhibit stress level practically unchanged up to reaching the yield point, and the strengthening effect is observed under further deformation, which is evident from an increase in σ_200%_ values up to 21%. This behavior indicates much lower interactions between two phases and suggests that for carbon nanotubes NC_02 and NC_03, they act as a lubricant for polymer chains rather than the reinforcement. Nanoparticles located in intermolecular regions increase the distance between chains, thus decreasing the molecular interaction strength, which is evident by a drop in *E* modulus values. However, they also facilitate macromolecules to slide one over another and their orientation, which results in stress increase during further deformation. It is symptomatic, that both nanotube types (NC_02 and NC_03) were “wet” modified. 

Practically no improvement in mechanical performance of PA/NC_01 composite was quite surprising in this study, since it was expected that commercially modified CNTs would bring even better strengthening effects when compared to untreated nanotubes as reported in [37]. This observation confirms once again, that surface functionalization may bring benefits only if it coexists with uniform distribution. Which is consistent with conclusions found in Chattarjee’s paper [31], where PA12 composites with CNT or graphene nanoplatelets, were examined. In those studies, the addition of oxidized CNTs not only failed in improvement of the stress level, but also drastically reduced the samples’ elongation, what was explained by poor nanofiller dispersion as well. The best results, in turn, were achieved for composites containing a mixture of nanocarbons with surfactants. 

**Figure 8:**
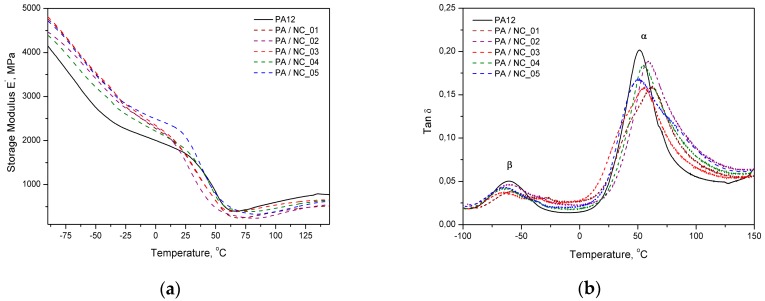
DMTA analysis of PA12/NC composites: (**a**) storage modulus and (**b**) loss factor.

Dynamic mechanical analysis also confirms the stiffening effect of carbon nanotubes by improved storage modulus of composites, but mainly below glass transition temperature (Figure 8a). Close to *T*_g_ region and above the modulus drops to the level of neat polymer or even lower. The same behavior was observed for PA12/CNT composites to laser sintering [56]. In turn, the values of composites’ loss factor (tanδ) (Figure 8b) are generally higher in the temperature range studied, excluding the temperature of α- and β-relaxation peaks (where α is associated to glass transition and β to movement of short chain segments), in which the reference reaches the highest values. It may be related to locating nanotubes in free volumes that hinders polymer chain vibrations and reduces the damping ability of composites at transition temperatures as compared to the unfilled matrix. However, dispersed carbon nanotubes also take a part in vibration transfer, but are not as flexible as macromolecules. It results in additional internal friction and generated energy is dissipated increasing tanδ values. Similar effects in tanδ characteristics were observed in the PA12/CNT composites with comparable CNT contents, obtained by in situ anionic ring opening polymerization via reactive extrusion, and extensively studied by DMTA [57]. It was explained as a restriction in polymer chains’ movement by chain grafted nanotubes. For higher CNT contents, however, the tanδ factor was increasing, and this effect was explained by broader molecular weight distribution of the polymer chains growing in the presence of nanotubes. Similar conditions accompanied the synthesis of composites investigated in this study. It should be also noticed that the β-relaxation effects in composites lose their intensity when compared to the neat polymer, what according to Ghislandi et al. [58] indicates a better nanofiller–polymer matrix interactions, as also observed for surface modified carbon nanofibres in PA12. 

**Figure 9:**
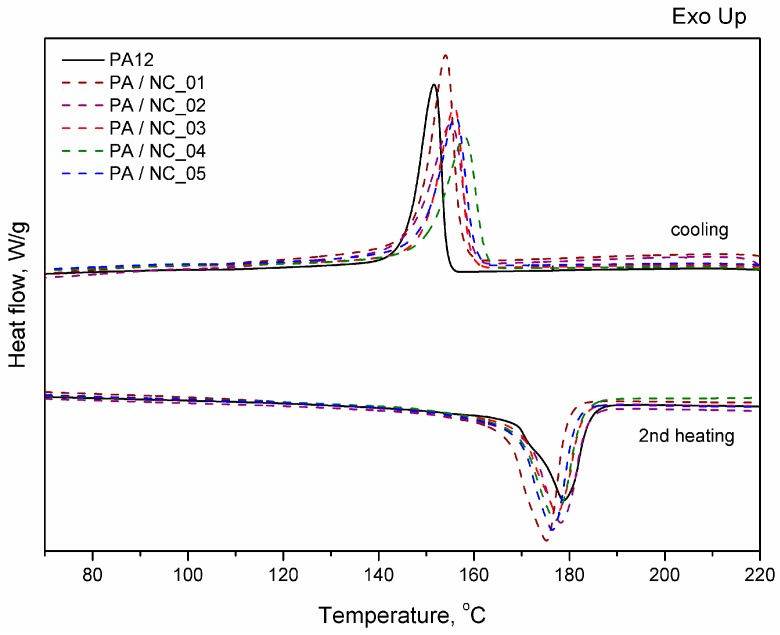
Differential scanning calorimeter (DSC) thermograms of PA12/NC composites.

**Figure 10:**
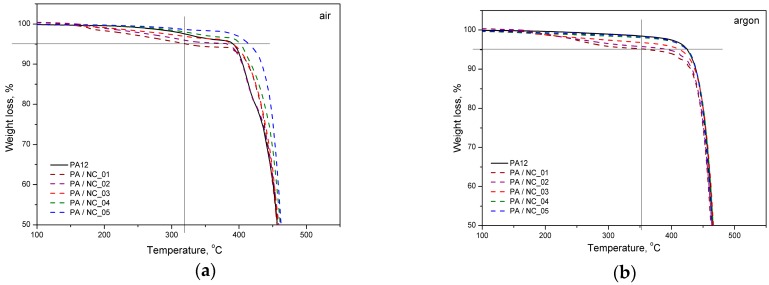
Thermal stability of PA/NC composites in atmosphere: (**a**) oxidative and (**b**) neutral.

Thermal analysis of PA-based composites brings further evidence for nanotube effect on polymer phase transitions and their microstructure. However, the procedure of CNT functionalization is of minor importance in this case. All investigated composites reveal a slight drop in melting temperature, while an increase in both crystallization temperature and crystallinity degree (Figure 9, Table 5). These observations are typical for nanocomposites and confirm the nucleating nature of nanoadditives [31,57,58]. The largest drop of T_m_ for PA/NC_01 suggests, that even if an increase in crystallinity degree is observed, the crystalline structure may be more defected or finer resulting in melting temperature lowering. This could be attributed to non-uniform dispersion of nanotubes in the polymer matrix rather than the functionalization procedure. If, however, the polymer’s crystallizability is considered, defined as the difference of Tm and Tc, and usually named as the degree of supercooling, all composites exhibit an overall increase in crystallization rate compared to homopolymer, regardless the type of CNT functionalization, and it is beneficial if materials processing is considered. On the other hand, the procedures of CNT chemical modification are gaining importance, when thermo-oxidative stability of composites is examined (Figure 10). From TGA thermograms and data collected in Table 5, it is clearly seen that again NC_04 and NC_05 composites reveal the best results. Basically, decomposition of polyamides proceeds in two stages with initial depolymerization at end-groups and chain cutting to formation of unsaturated nitriles and alkenes. The reactions are even accelerated in the presence of oxygen [59]. The visible decrease in thermal stability of composites containing NC_01, NC_02, and NC_03 nanotubes with oxygen-containing groups indicates that they had not taken a part in polymerization to react with polymer chains, but remained still reactive in composites promoting and contributing to polymer decomposition at high temperature and oxidizing atmosphere. It is also proved by the results of thermal stability in inert gas atmosphere. Undoubtedly in this case decomposition is induced mainly by a higher concentration of oxygen-containing groups attached to CNTs if compared to the reference sample. These results bring another strong evidence for enhanced molecular interactions in PA/NC_04 and PA/NC_05 composites.

Generally speaking, all laboratory-treated CNT were characterized by high surface roughness, which suggests that in all cases their graphite structure suffered due to the chemical treatment conditions, regardless of which procedure was used. However, NC_02 reveals the most defected structure combined with the highest value of ID/IG Raman intensity ratio, which proves its low quality and results in relatively poor PA/NC_02 performance, despite a high concentration of oxygen on the surface, mainly in the form of carboxyl functional groups. It leads to the conclusion that simple treatment of highly oxidizing acid (“wet” method) is the most harmful and destructive for nanocarbon structures, and practically eliminates CNT as reinforcement for polymer composites. Although the characterization parameters of NC_03 nanotubes are higher and suggest more effective surface functionalization, PA/NC_03 properties are only slightly improved if compared to a neat polymer. The reason might be a lower concentration of oxygen functional groups achieved on CNT surface in comparison with a concentration obtained for the material treated with nitric acid (NC_02) (Table 2). According to XPS results, hydroxyl groups dominate in this sample, which limits interphase interactions in composites to physical only. 

Two nanotube samples, i.e., NC_04 and NC_05, giving the best results in composite performance, differ from others, but also with one another, in their surface composition. NC_05 is characterized by the presence of aluminum atoms incorporated during the modification step, detected by XPS. However, as described above, due to a significant charging of NC_05 surface its detailed characterization was not possible. Despite this, carboxyl groups in the sample NC_05 are expected likewise in NC_04 sample. Moreover, the common feature for NC_04 and NC_05 is, that both were functionalized in the “gas” phase with chlorination and subsequent ammonia treatment. What is more, even though the last step was different for both samples the obtained results confirmed that “gaseous” modification is more gentle for nanotube structure and most efficient in functional groups’ concentration. It is also beneficial for PA12/CNT composites by improving their mechanical performance and thermo-oxidative stability. For comparison Roy et al. [60] used multi-walled carbon nanotubes modified by radiofrequency oxygen plus plasma treatment, which is considered as much less destructive and harmful for graphitic structure, to prepare PA12 composites by melt mixing. Considering the mechanical parameters for composites containing a comparable CNT content (0.35 versus 0.4 wt % of plasma treated nanotubes) the materials investigated in this study reveal slightly better effects of improvement, i.e., maximum increase of stress up to 26% versus ca. 16%, *E* modulus up to 39% versus 18%, and relatively high strain at break of ca. 300% versus ca. 130%. It should be noticed however, that in our case the tests were performed on injection molded bulk samples, whilst Roy’s composites had a form of films, what explains different shapes of stress–strain curves, and significantly higher E modulus values of our PA/NC materials (actually the E values were the highest if compared to the results reported in [57,60]). At the same time the thermal and structural parameters of two kinds of composites are similar, what brings another evidence, that in our study the composite performance results from inter-molecular interactions between filler and polymer matrix improved by CNT surface functionalization. 

The most surprising and meaningful results were obtained for commercially functionalized carbon nanotube filled composites (PA/NC_01). This material was characterized by a high concentration of oxygen (7.6 atomic %), mainly in the form of carboxyl functional groups. It brings unquestionable evidence that the effect of CNT modification may be severely diminished due to the lack of nanotube uniform distribution in the polymer matrix. 

## 4. Conclusions

The purpose of this study was a qualitative and quantitative evaluation of multi-walled carbon nanotubes functionalized with oxygen containing reactive groups achieved by different procedures of chemical treatment. Simple strong oxidative acid treatment was employed to NC_02 sample or chlorination and subsequent chloroacetic acid treatment for NC_03 sample. The samples NC_04 and NC_05 were subjected to chlorination and ammonia in gaseous atmosphere with small differences in after-ammonia treatment. Commercial COOH– functionalized carbon nanotubes were also examined to compare with those that are laboratory modified (NC_01). The effect of CNT functionalization was also evaluated based on the improvement in mechanical and thermal properties of polyamide 12 composites prepared by in situ polymerization. The studies prove that a high concentration of oxygen-containing functional groups on nanotube surface does not contribute to satisfactory improvement in composite performance if graphene structure of a nanofiller is highly defected. That was observed for CNTs after strongly oxidizing “wet” treatment. Indeed, the best effects were achieved for composites containing nanotubes modified under mild conditions, which seems to be a compromise between morphology and surface chemical structure. The case of commercially functionalized carbon nanotubes shows that high quality material does not guarantee expected effects if there is insufficient care in bulk CNT powder preparation.

## Figures and Tables

**Table 1:** The intensity ratio of D to G band intensity ratio (ID/IG) calculated for pristine and functionalized carbon nanotubes.

Sample	NC	NC_01	NC_02	NC_03	NC_04	NC_05
**ID/IG**	1.55	1.76	1.87	1.60	1.67	1.72

**Table 2:** Surface composition of carbon nanotubes.

Sample	Carbon	Oxygen	Chlorine	Nitrogen	Aluminum
	atomic %				
NC_01	92.7	7.3	-	data	-
NC_02	93.6	6.4	-	traces	-
NC_03	96.5	3.0	0.5	-	-
NC_04	96.9	2.5	0.6	traces	-
NC_05	75.7	13.2	3.7	-	7.4

**Table 3:** Fractional composition of chemical states of oxygen atoms observed on the surface of carbon nanotubes.

Sample	C=O	C–O	H_2_O	Satellite
	% of Total O 1s Signal Intensity			
NC_01	56.7	39.1	4.2	-
NC_02	35.7	54.0	10.3	-
NC_03	8.2	86.4	5.4	-
NC_04	22.2	43.6	20.4	13.8

**Table 4:** Molecular weights and mechanical parameters of neat PA12 and PA based composites.

Sample	*M*_w_ × 10^3^ [g/mol]	σ_y_ [MPa]	σ_b_ [MPa]	σ_200%_ [MPa]	ε_b_ [%]	*E* [MPa]
PA neat	29.3±0.2	34.7 ± 0.16	60.7 ± 4.67	36.1 ± 0.26	347 ± 21	878 ± 50
PA/NC_01	29.6±0.3	30.1 ± 0.61	51.5 ± 3.15	38.2 ± 0.19	309 ± 15	742 ± 26
PA/NC_02	28.5±0.2	33.4 ± 0.22	53.1 ± 6.40	43.7 ± 0.16	291 ± 20	757 ± 15
PA/NC_03	29.1±0.2	34.8 ± 0.19	62.3 ± 1.27	43.7 ± 0.19	308 ± 5	739 ± 28
PA/NC_04	27.7±0.3	42.4 ± 0.29	54.2 ± 2.06	45.8 ± 0.21	272 ± 19	1177 ± 72
PA/NC_05	27.1±0.2	43.8 ± 0.16	57.0 ± 2.40	46.6 ± 0.20	284 ± 13	1220 ± 61

σ_y_; yield stress, σ_b_; stress at break, σ_200%_; stress at 200% of strain, ε_b_; strain at break, and *E*; Young’s modulus, the values are the average from at least six tests for each sample.

**Table 5:** Thermal properties of neat PA12 and PA based composites.

Sample	DSC			DSC		Thermal Stability			
	Heating Cycle			Cooling Cycle		Air		argon	
	*T*_m_ [°C]	*T*_g_ [°C]	*X*_c_ [%]	*T*_c max_ [°C]	Δ*T* [°C]	*T*_5%_ [°C]	*T*_max_ [°C]	*T*_5%_ [°C]	*T*_max_ [°C]
PA12	179	46	27	152	27	392	461	425	472
PA/NC_01	173	50	33	152	21	323	455	363	466
PA/NC_02	177	49	34	155	22	382	471	394	469
PA/NC_03	177	49	30	156	21	392	465	415	472
PA/NC_04	178	48	29	158	20	401	465	424	471
PA/NC_05	176	47	30	156	20	416	469	425	471

Tm; melting temperature, *T*_g_; glass transition temperature, *X*_c_; crystallinity degree, *T*_c_; crystallization temperature, Δ*T* = *T*_m_; *T*_c_ degree of supercooling, and *T*_5%_ and *T*_max_; temperature of 5% and maximum decomposition of polymer matrix, respectively. The standard deviation for DSC and TGA measurements ±1 °C.
